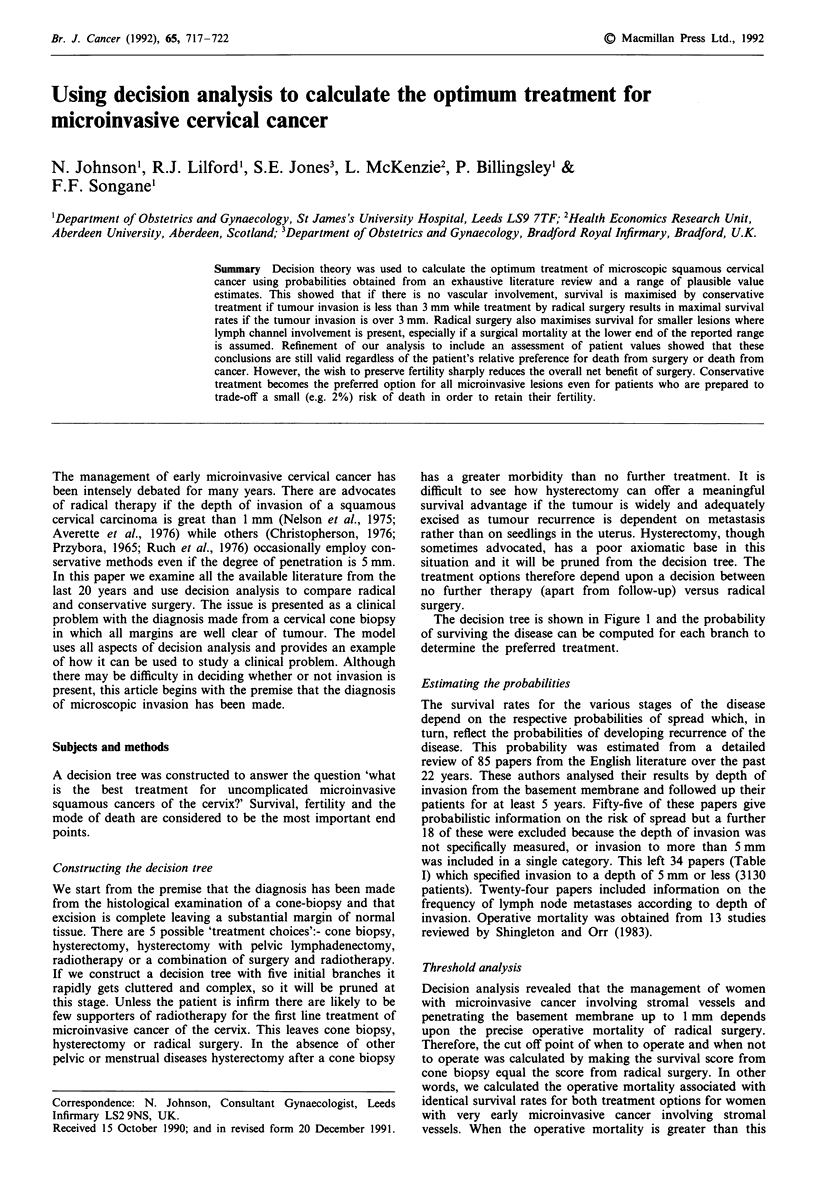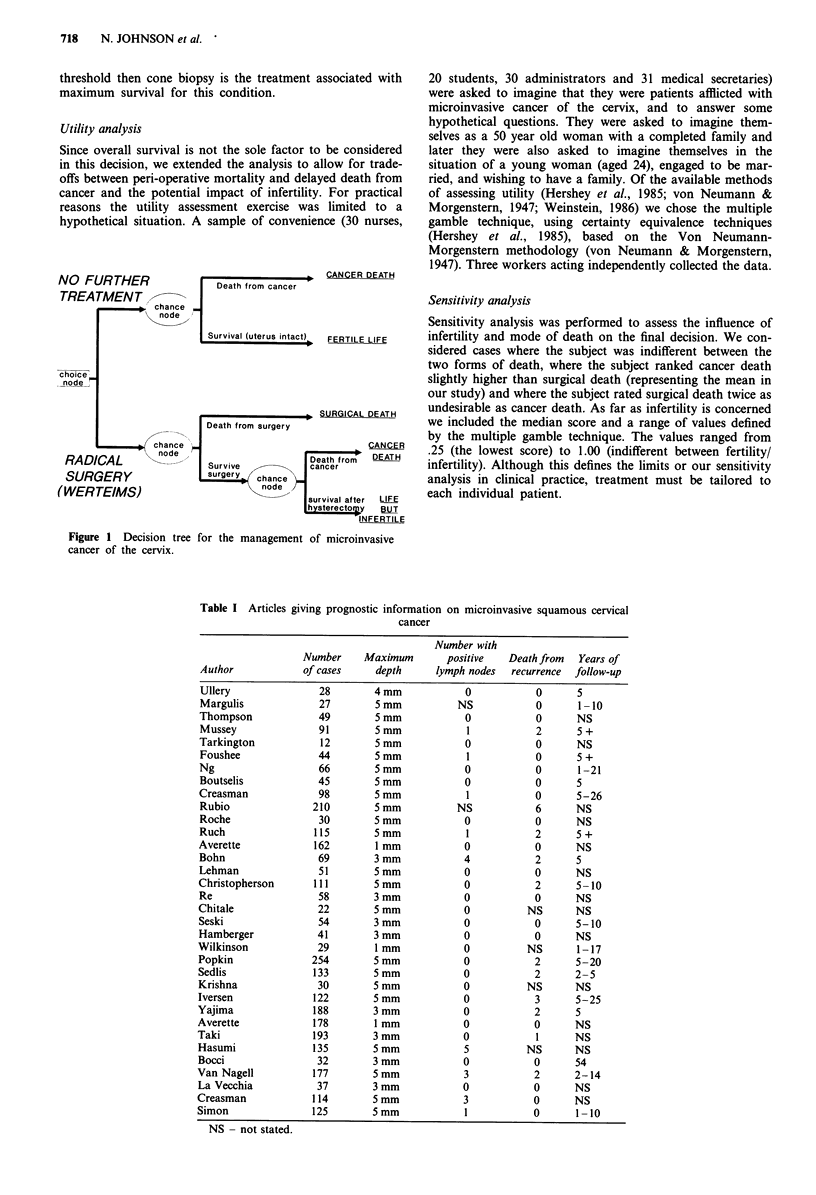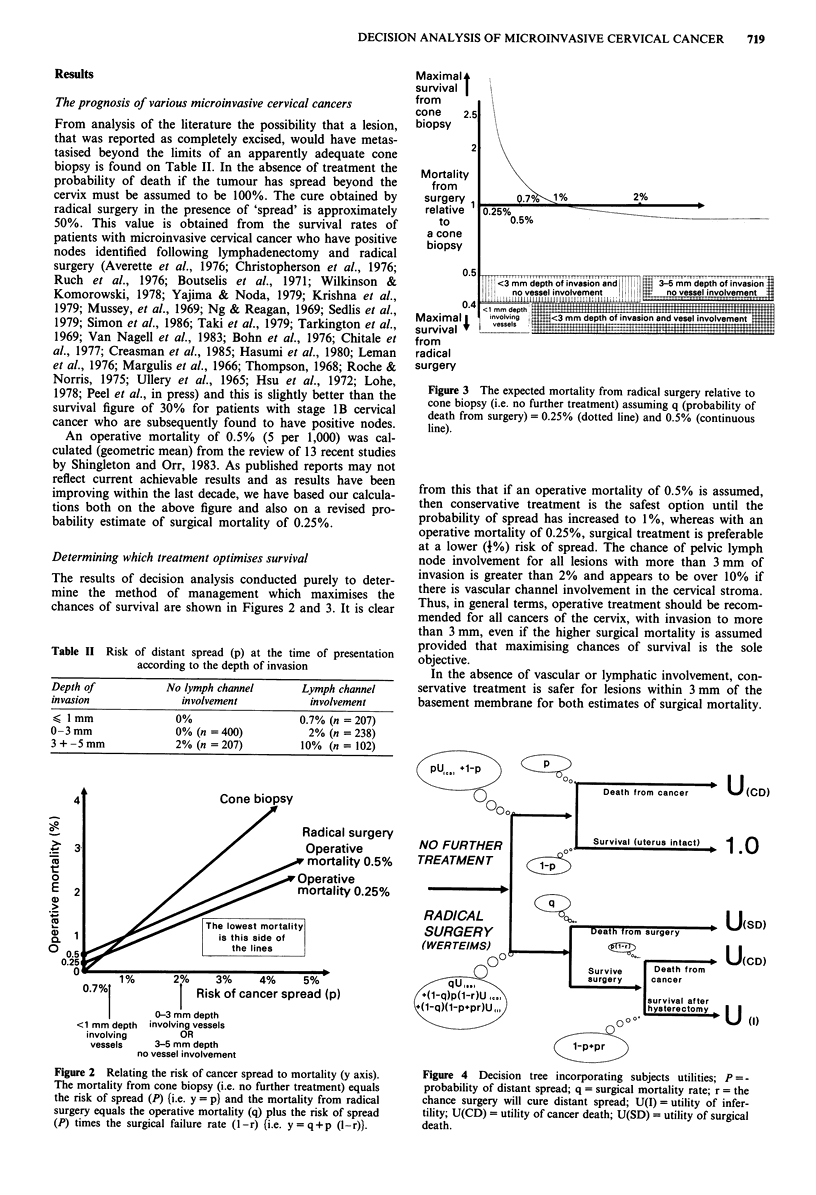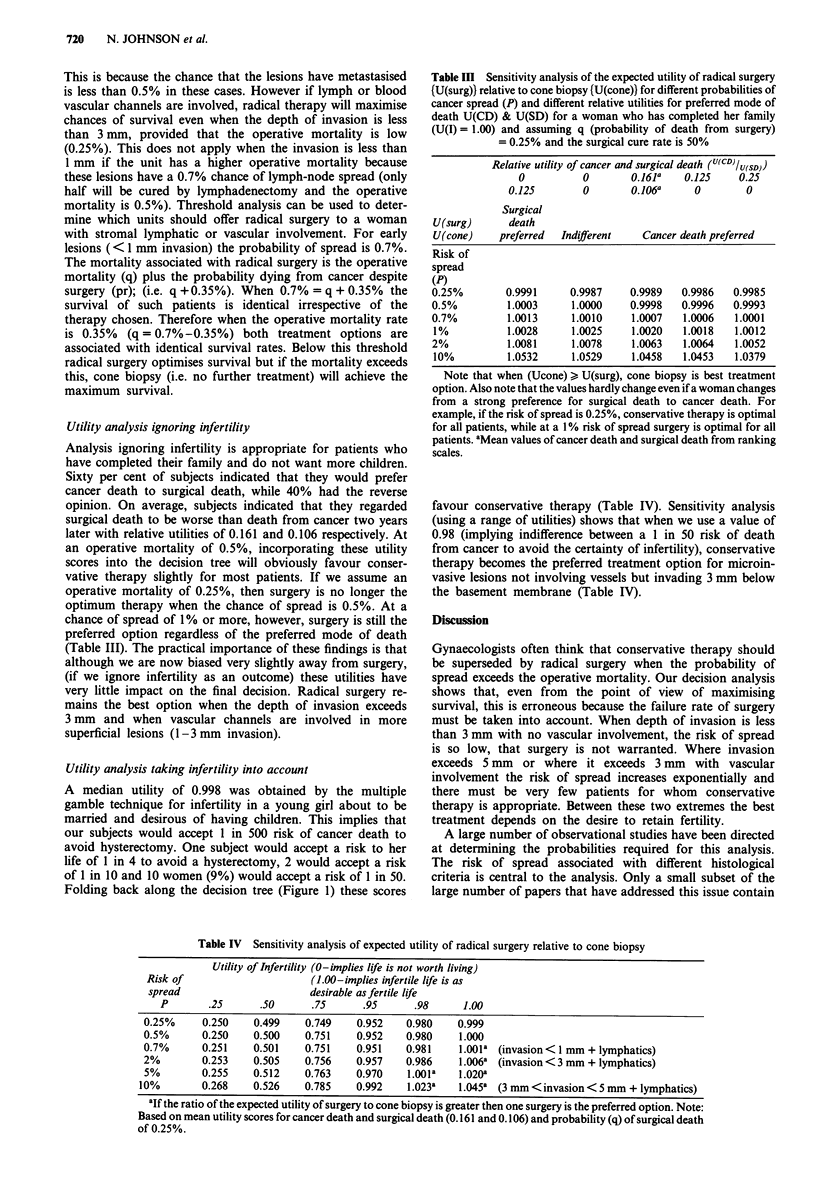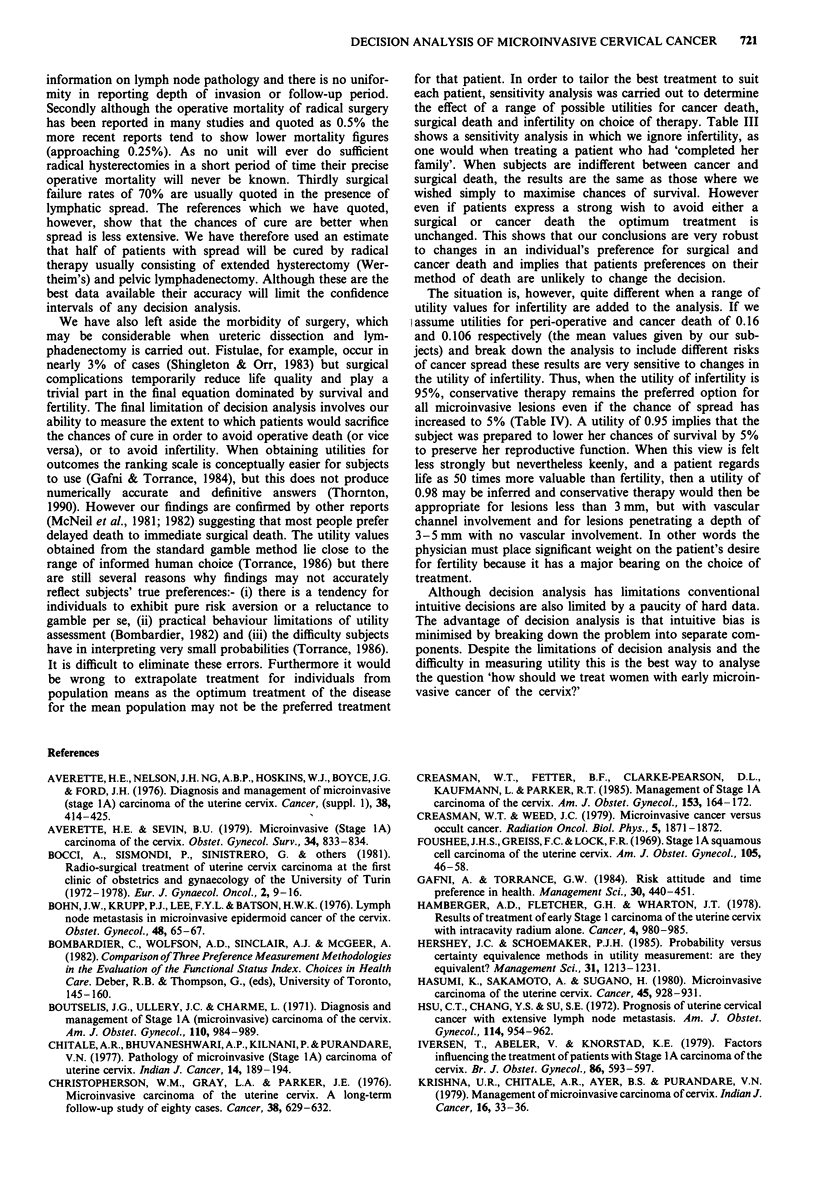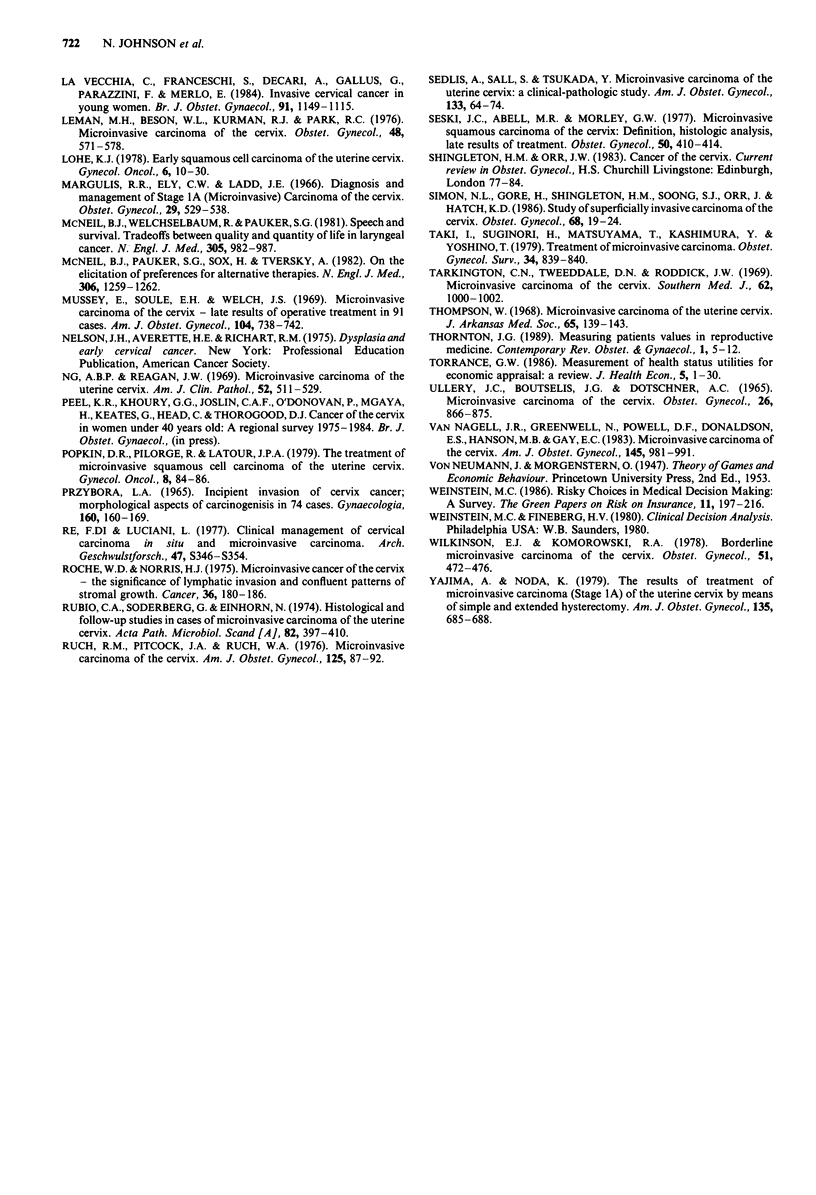# Using decision analysis to calculate the optimum treatment for microinvasive cervical cancer.

**DOI:** 10.1038/bjc.1992.151

**Published:** 1992-05

**Authors:** N. Johnson, R. J. Lilford, S. E. Jones, L. McKenzie, P. Billingsley, F. F. Songane

**Affiliations:** Department of Obstetrics and Gynaecology, St James's University Hospital, Leeds, U.K.

## Abstract

Decision theory was used to calculate the optimum treatment of microscopic squamous cervical cancer using probabilities obtained from an exhaustive literature review and a range of plausible value estimates. This showed that if there is no vascular involvement, survival is maximised by conservative treatment if tumour invasion is less than 3 mm while treatment by radical surgery results in maximal survival rates if the tumour invasion is over 3 mm. Radical surgery also maximises survival for smaller lesions where lymph channel involvement is present, especially if a surgical mortality at the lower end of the reported range is assumed. Refinement of our analysis to include an assessment of patient values showed that these conclusions are still valid regardless of the patient's relative preference for death from surgery or death from cancer. However, the wish to preserve fertility sharply reduces the overall net benefit of surgery. Conservative treatment becomes the preferred option for all microinvasive lesions even for patients who are prepared to trade-off a small (e.g. 2%) risk of death in order to retain their fertility.


Br. J. Cancer (1992), 65, 717-722 © Macmillan Press Ltd., 1992

### Using decision analysis to calculate the optimum treatment for microinvasive cervical cancer

N. Johnson[1], R.J. Lilford[1], S.E. Jones[3], L. McKenzie[2], P. Billingsley[1] & F.F. Songane[1]

[1]*Department of Obstetrics and Gynaecology, St James's University Hospital, Leeds LS9 7TF;* [2]*Health Economics Research Unit, Aberdeen University, Aberdeen, Scotland;* [3]*Department of Obstetrics and Gynaecology, Bradford Royal Infirmary, Bradford, U.K.*

**Summary** Decision theory was used to calculate the optimum treatment of microscopic squamous cervical cancer using probabilities obtained from an exhaustive literature review and a range of plausible value estimates. This showed that if there is no vascular involvement, survival is maximised by conservative treatment if tumour invasion is less than 3 mm while treatment by radical surgery results in maximal survival rates if the tumour invasion is over 3 mm. Radical surgery also maximises survival for smaller lesions where lymph channel involvement is present, especially if a surgical mortality at the lower end of the reported range is assumed. Refinement of our analysis to include an assessment of patient values showed that these conclusions are still valid regardless of the patient's relative preference for death from surgery or death from cancer. However, the wish to preserve fertility sharply reduces the overall net benefit of surgery. Conservative treatment becomes the preferred option for all microinvasive lesions even for patients who are prepared to trade-off a small (e.g. 2%) risk of death in order to retain their fertility.

The management of early microinvasive cervical cancer has been intensely debated for many years. There are advocates of radical therapy if the depth of invasion of a squamous cervical carcinoma is great than 1 mm (Nelson *et al.*, 1975; Averette *et al.*, 1976) while others (Christopherson, 1976; Przybora, 1965; Ruch *et al.*, 1976) occasionally employ conservative methods even if the degree of penetration is 5 mm. In this paper we examine all the available literature from the last 20 years and use decision analysis to compare radical and conservative surgery. The issue is presented as a clinical problem with the diagnosis made from a cervical cone biopsy in which all margins are well clear of tumour. The model uses all aspects of decision analysis and provides an example of how it can be used to study a clinical problem. Although there may be difficulty in deciding whether or not invasion is present, this article begins with the premise that the diagnosis of microscopic invasion has been made.

### Subjects and methods

A decision tree was constructed to answer the question 'what is the best treatment for uncomplicated microinvasive squamous cancers of the cervix?' Survival, fertility and the mode of death are considered to be the most important end points.

### *Constructing the decision tree*

We start from the premise that the diagnosis has been made from the histological examination of a cone-biopsy and that excision is complete leaving a substantial margin of normal tissue. There are 5 possible 'treatment choices':- cone biopsy, hysterectomy, hysterectomy with pelvic lymphadenectomy, radiotherapy or a combination of surgery and radiotherapy. If we construct a decision tree with five initial branches it rapidly gets cluttered and complex, so it will be pruned at this stage. Unless the patient is infirm there are likely to be few supporters of radiotherapy for the first line treatment of microinvasive cancer of the cervix. This leaves cone biopsy, hysterectomy or radical surgery. In the absence of other pelvic or menstrual diseases hysterectomy after a cone biopsy has a greater morbidity than no further treatment. It is difficult to see how hysterectomy can offer a meaningful survival advantage if the tumour is widely and adequately excised as tumour recurrence is dependent on metastasis rather than on seedlings in the uterus. Hysterectomy, though sometimes advocated, has a poor axiomatic base in this situation and it will be pruned from the decision tree. The treatment options therefore depend upon a decision between no further therapy (apart from follow-up) versus radical surgery.

The decision tree is shown in Figure 1 and the probability of surviving the disease can be computed for each branch to determine the preferred treatment.

### *Estimating the probabilities*

The survival rates for the various stages of the disease depend on the respective probabilities of spread which, in turn, reflect the probabilities of developing recurrence of the disease. This probability was estimated from a detailed review of 85 papers from the English literature over the past 22 years. These authors analysed their results by depth of invasion from the basement membrane and followed up their patients for at least 5 years. Fifty-five of these papers give probabilistic information on the risk of spread but a further 18 of these were excluded because the depth of invasion was not specifically measured, or invasion to more than 5 mm was included in a single category. This left 34 papers (Table I) which specified invasion to a depth of 5 mm or less (3130 patients). Twenty-four papers included information on the frequency of lymph node metastases according to depth of invasion. Operative mortality was obtained from 13 studies reviewed by Shingleton and Orr (1983).

### *Threshold analysis*

Decision analysis revealed that the management of women with microinvasive cancer involving stromal vessels and penetrating the basement membrane up to 1 mm depends upon the precise operative mortality of radical surgery. Therefore, the cut off point of when to operate and when not to operate was calculated by making the survival score from cone biopsy equal the score from radical surgery. In other words, we calculated the operative mortality associated with identical survival rates for both treatment options for women with very early microinvasive cancer involving stromal vessels. When the operative mortality is greater than this

Correspondence: N. Johnson, Consultant Gynaecologist, Leeds Infirmary LS2 9NS, UK.
Received 15 October 1990; and in revised form 20 December 1991.

threshold then cone biopsy is the treatment associated with maximum survival for this condition.

### *Utility analysis*

Since overall survival is not the sole factor to be considered in this decision, we extended the analysis to allow for trade-offs between peri-operative mortality and delayed death from cancer and the potential impact of infertility. For practical reasons the utility assessment exercise was limited to a hypothetical situation. A sample of convenience (30 nurses, 20 students, 30 administrators and 31 medical secretaries) were asked to imagine that they were patients afflicted with microinvasive cancer of the cervix, and to answer some hypothetical questions. They were asked to imagine themselves as a 50 year old woman with a completed family and later they were also asked to imagine themselves in the situation of a young woman (aged 24), engaged to be married, and wishing to have a family. Of the available methods of assessing utility (Hershey *et al.*, 1985; von Neumann & Morgenstern, 1947; Weinstein, 1986) we chose the multiple gamble technique, using certainty equivalence techniques (Hershey *et al.*, 1985), based on the Von Neumann-Morgenstern methodology (von Neumann & Morgenstern, 1947). Three workers acting independently collected the data.

**Figure 1** Decision tree for the management of microinvasive cancer of the cervix.

### *Sensitivity analysis*

Sensitivity analysis was performed to assess the influence of infertility and mode of death on the final decision. We considered cases where the subject was indifferent between the two forms of death, where the subject ranked cancer death slightly higher than surgical death (representing the mean in our study) and where the subject rated surgical death twice as undesirable as cancer death. As far as infertility is concerned we included the median score and a range of values defined by the multiple gamble technique. The values ranged from .25 (the lowest score) to 1.00 (indifferent between fertility/infertility). Although this defines the limits or our sensitivity analysis in clinical practice, treatment must be tailored to each individual patient.

**Table I** Articles giving prognostic information on microinvasive squamous cervical cancer

| *Author* | *Number of cases* | *Maximum depth* | *Number with positive lymph nodes* | *Death from recurrence* | *Years of follow-up* |
|---|---|---|---|---|---|
| Ullery | 28 | 4 mm | 0 | 0 | 5 |
| Margulis | 27 | 5 mm | NS | 0 | 1–10 |
| Thompson | 49 | 5 mm | 0 | 0 | NS |
| Mussey | 91 | 5 mm | 1 | 2 | 5+ |
| Tarkington | 12 | 5 mm | 0 | 0 | NS |
| Foushee | 44 | 5 mm | 1 | 0 | 5+ |
| Ng | 66 | 5 mm | 0 | 0 | 1–21 |
| Boutselis | 45 | 5 mm | 0 | 0 | 5 |
| Creasman | 98 | 5 mm | 1 | 0 | 5–26 |
| Rubio | 210 | 5 mm | NS | 6 | NS |
| Roche | 30 | 5 mm | 0 | 0 | NS |
| Ruch | 115 | 5 mm | 1 | 2 | 5+ |
| Averette | 162 | 1 mm | 0 | 0 | NS |
| Bohn | 69 | 3 mm | 4 | 2 | 5 |
| Lehman | 51 | 5 mm | 0 | 0 | NS |
| Christopherson | 111 | 5 mm | 0 | 2 | 5–10 |
| Re | 58 | 3 mm | 0 | 0 | NS |
| Chitale | 22 | 5 mm | 0 | NS | NS |
| Seski | 54 | 3 mm | 0 | 0 | 5–10 |
| Hamberger | 41 | 3 mm | 0 | 0 | NS |
| Wilkinson | 29 | 1 mm | 0 | NS | 1–17 |
| Popkin | 254 | 5 mm | 0 | 2 | 5–20 |
| Sedlis | 133 | 5 mm | 0 | 2 | 2–5 |
| Krishna | 30 | 5 mm | 0 | NS | NS |
| Iversen | 122 | 5 mm | 0 | 3 | 5–25 |
| Yajima | 188 | 3 mm | 0 | 2 | 5 |
| Averette | 178 | 1 mm | 0 | 0 | NS |
| Taki | 193 | 3 mm | 0 | 1 | NS |
| Hasumi | 135 | 5 mm | 5 | NS | NS |
| Bocci | 32 | 3 mm | 0 | 0 | 54 |
| Van Nagell | 177 | 5 mm | 3 | 2 | 2–14 |
| La Vecchia | 37 | 3 mm | 0 | 0 | NS |
| Creasman | 114 | 5 mm | 3 | 0 | NS |
| Simon | 125 | 5 mm | 1 | 0 | 1–10 |

NS – not stated.

### Results

### *The prognosis of various microinvasive cervical cancers*

From analysis of the literature the possibility that a lesion, that was reported as completely excised, would have metastasised beyond the limits of an apparently adequate cone biopsy is found on Table II. In the absence of treatment the probability of death if the tumour has spread beyond the cervix must be assumed to be 100%. The cure obtained by radical surgery in the presence of 'spread' is approximately 50%. This value is obtained from the survival rates of patients with microinvasive cervical cancer who have positive nodes identified following lymphadenectomy and radical surgery (Averette *et al.*, 1976; Christopherson *et al.*, 1976; Ruch *et al.*, 1976; Boutselis *et al.*, 1971; Wilkinson & Komorowski, 1978; Yajima & Noda, 1979; Krishna *et al.*, 1979; Mussey, *et al.*, 1969; Ng & Reagan, 1969; Sedlis *et al.*, 1979; Simon *et al.*, 1986; Taki *et al.*, 1979; Tarkington *et al.*, 1969; Van Nagell *et al.*, 1983; Bohn *et al.*, 1976; Chitale *et al.*, 1977; Creasman *et al.*, 1985; Hasumi *et al.*, 1980; Leman *et al.*, 1976; Margulis *et al.*, 1966; Thompson, 1968; Roche & Norris, 1975; Ullery *et al.*, 1965; Hsu *et al.*, 1972; Lohe, 1978; Peel *et al.*, in press) and this is slightly better than the survival figure of 30% for patients with stage 1B cervical cancer who are subsequently found to have positive nodes.

An operative mortality of 0.5% (5 per 1,000) was calculated (geometric mean) from the review of 13 recent studies by Shingleton and Orr, 1983. As published reports may not reflect current achievable results and as results have been improving within the last decade, we have based our calculations both on the above figure and also on a revised probability estimate of surgical mortality of 0.25%.

### *Determining which treatment optimises survival*

The results of decision analysis conducted purely to determine the method of management which maximises the chances of survival are shown in Figures 2 and 3. It is clear from this that if an operative mortality of 0.5% is assumed, then conservative treatment is the safest option until the probability of spread has increased to 1%, whereas with an operative mortality of 0.25%, surgical treatment is preferable at a lower (½%) risk of spread. The chance of pelvic lymph node involvement for all lesions with more than 3 mm of invasion is greater than 2% and appears to be over 10% if there is vascular channel involvement in the cervical stroma. Thus, in general terms, operative treatment should be recommended for all cancers of the cervix, with invasion to more than 3 mm, even if the higher surgical mortality is assumed provided that maximising chances of survival is the sole objective.

In the absence of vascular or lymphatic involvement, conservative treatment is safer for lesions within 3 mm of the basement membrane for both estimates of surgical mortality.

**Table II** Risk of distant spread (p) at the time of presentation according to the depth of invasion

| *Depth of invasion* | *No lymph channel involvement* | *Lymph channel involvement* |
|---|---|---|
| ⩽ 1 mm | 0% | 0.7% ($n$ = 207) |
| 0–3 mm | 0% ($n$ = 400) | 2% ($n$ = 238) |
| 3+–5 mm | 2% ($n$ = 207) | 10% ($n$ = 102) |

**Figure 2** Relating the risk of cancer spread to mortality (y axis). The mortality from cone biopsy (i.e. no further treatment) equals the risk of spread ($P$) {i.e. y = p} and the mortality from radical surgery equals the operative mortality (q) plus the risk of spread ($P$) times the surgical failure rate (1−r) {i.e. y = q+p (1−r)}.

**Figure 3** The expected mortality from radical surgery relative to cone biopsy (i.e. no further treatment) assuming q (probability of death from surgery) = 0.25% (dotted line) and 0.5% (continuous line).

**Figure 4** Decision tree incorporating subjects utilities; $P$ = probability of distant spread; q = surgical mortality rate; r = the chance surgery will cure distant spread; U(I) = utility of infertility; U(CD) = utility of cancer death; U(SD) = utility of surgical death.

This is because the chance that the lesions have metastasised is less than 0.5% in these cases. However if lymph or blood vascular channels are involved, radical therapy will maximise chances of survival even when the depth of invasion is less than 3 mm, provided that the operative mortality is low (0.25%). This does not apply when the invasion is less than 1 mm if the unit has a higher operative mortality because these lesions have a 0.7% chance of lymph-node spread (only half will be cured by lymphadenectomy and the operative mortality is 0.5%). Threshold analysis can be used to determine which units should offer radical surgery to a woman with stromal lymphatic or vascular involvement. For early lesions (< 1 mm invasion) the probability of spread is 0.7%. The mortality associated with radical surgery is the operative mortality (q) plus the probability dying from cancer despite surgery (pr); (i.e. q + 0.35%). When 0.7% = q + 0.35% the survival of such patients is identical irrespective of the therapy chosen. Therefore when the operative mortality rate is 0.35% (q = 0.7% − 0.35%) both treatment options are associated with identical survival rates. Below this threshold radical surgery optimises survival but if the mortality exceeds this, cone biopsy (i.e. no further treatment) will achieve the maximum survival.

### *Utility analysis ignoring infertility*

Analysis ignoring infertility is appropriate for patients who have completed their family and do not want more children. Sixty per cent of subjects indicated that they would prefer cancer death to surgical death, while 40% had the reverse opinion. On average, subjects indicated that they regarded surgical death to be worse than death from cancer two years later with relative utilities of 0.161 and 0.106 respectively. At an operative mortality of 0.5%, incorporating these utility scores into the decision tree will obviously favour conservative therapy slightly for most patients. If we assume an operative mortality of 0.25%, then surgery is no longer the optimum therapy when the chance of spread is 0.5%. At a chance of spread of 1% or more, however, surgery is still the preferred option regardless of the preferred mode of death (Table III). The practical importance of these findings is that although we are now biased very slightly away from surgery, (if we ignore infertility as an outcome) these utilities have very little impact on the final decision. Radical surgery remains the best option when the depth of invasion exceeds 3 mm and when vascular channels are involved in more superficial lesions (1–3 mm invasion).

### *Utility analysis taking infertility into account*

A median utility of 0.998 was obtained by the multiple gamble technique for infertility in a young girl about to be married and desirous of having children. This implies that our subjects would accept 1 in 500 risk of cancer death to avoid hysterectomy. One subject would accept a risk to her life of 1 in 4 to avoid a hysterectomy, 2 would accept a risk of 1 in 10 and 10 women (9%) would accept a risk of 1 in 50. Folding back along the decision tree (Figure 1) these scores favour conservative therapy (Table IV). Sensitivity analysis (using a range of utilities) shows that when we use a value of 0.98 (implying indifference between a 1 in 50 risk of death from cancer to avoid the certainty of infertility), conservative therapy becomes the preferred treatment option for microinvasive lesions not involving vessels but invading 3 mm below the basement membrane (Table IV).

**Table III** Sensitivity analysis of the expected utility of radical surgery {U(surg)} relative to cone biopsy {U(cone)} for different probabilities of cancer spread (*P*) and different relative utilities for preferred mode of death U(CD) & U(SD) for a woman who has completed her family (U(I) = 1.00) and assuming q (probability of death from surgery) = 0.25% and the surgical cure rate is 50%

| | *Relative utility of cancer and surgical death* ($^{U(CD)}/_{U(SD)}$) | | | | |
|---|---|---|---|---|---|
| | *0* | *0* | *0.161*[a] | *0.125* | *0.25* |
| | *0.125* | *0* | *0.106*[a] | *0* | *0* |
| *U(surg)* / *U(cone)* | *Surgical death preferred* | *Indifferent* | *Cancer death preferred* | | |
| Risk of spread (*P*) | | | | | |
| 0.25% | 0.9991 | 0.9987 | 0.9989 | 0.9986 | 0.9985 |
| 0.5% | 1.0003 | 1.0000 | 0.9998 | 0.9996 | 0.9993 |
| 0.7% | 1.0013 | 1.0010 | 1.0007 | 1.0006 | 1.0001 |
| 1% | 1.0028 | 1.0025 | 1.0020 | 1.0018 | 1.0012 |
| 2% | 1.0081 | 1.0078 | 1.0063 | 1.0064 | 1.0052 |
| 10% | 1.0532 | 1.0529 | 1.0458 | 1.0453 | 1.0379 |

Note that when (Ucone) ⩾ U(surg), cone biopsy is best treatment option. Also note that the values hardly change even if a woman changes from a strong preference for surgical death to cancer death. For example, if the risk of spread is 0.25%, conservative therapy is optimal for all patients, while at a 1% risk of spread surgery is optimal for all patients. [a]Mean values of cancer death and surgical death from ranking scales.

### Discussion

Gynaecologists often think that conservative therapy should be superseded by radical surgery when the probability of spread exceeds the operative mortality. Our decision analysis shows that, even from the point of view of maximising survival, this is erroneous because the failure rate of surgery must be taken into account. When depth of invasion is less than 3 mm with no vascular involvement, the risk of spread is so low, that surgery is not warranted. Where invasion exceeds 5 mm or where it exceeds 3 mm with vascular involvement the risk of spread increases exponentially and there must be very few patients for whom conservative therapy is appropriate. Between these two extremes the best treatment depends on the desire to retain fertility.

A large number of observational studies have been directed at determining the probabilities required for this analysis. The risk of spread associated with different histological criteria is central to the analysis. Only a small subset of the large number of papers that have addressed this issue contain

**Table IV** Sensitivity analysis of expected utility of radical surgery relative to cone biopsy

| *Risk of spread P* | *Utility of Infertility (0–implies life is not worth living) (1.00–implies infertile life is as desirable as fertile life)* | | | | | | |
|---|---|---|---|---|---|---|---|
| | *.25* | *.50* | *.75* | *.95* | *.98* | *1.00* | |
| 0.25% | 0.250 | 0.499 | 0.749 | 0.952 | 0.980 | 0.999 | |
| 0.5% | 0.250 | 0.500 | 0.751 | 0.952 | 0.980 | 1.000 | |
| 0.7% | 0.251 | 0.501 | 0.751 | 0.951 | 0.981 | 1.001[a] | (invasion < 1 mm + lymphatics) |
| 2% | 0.253 | 0.505 | 0.756 | 0.957 | 0.986 | 1.006[a] | (invasion < 3 mm + lymphatics) |
| 5% | 0.255 | 0.512 | 0.763 | 0.970 | 1.001[a] | 1.020[a] | |
| 10% | 0.268 | 0.526 | 0.785 | 0.992 | 1.023[a] | 1.045[a] | (3 mm < invasion < 5 mm + lymphatics) |

[a]If the ratio of the expected utility of surgery to cone biopsy is greater then one surgery is the preferred option. Note: Based on mean utility scores for cancer death and surgical death (0.161 and 0.106) and probability (q) of surgical death of 0.25%.

information on lymph node pathology and there is no uniformity in reporting depth of invasion or follow-up period. Secondly although the operative mortality of radical surgery has been reported in many studies and quoted as 0.5% the more recent reports tend to show lower mortality figures (approaching 0.25%). As no unit will ever do sufficient radical hysterectomies in a short period of time their precise operative mortality will never be known. Thirdly surgical failure rates of 70% are usually quoted in the presence of lymphatic spread. The references which we have quoted, however, show that the chances of cure are better when spread is less extensive. We have therefore used an estimate that half of patients with spread will be cured by radical therapy usually consisting of extended hysterectomy (Wertheim's) and pelvic lymphadenectomy. Although these are the best data available their accuracy will limit the confidence intervals of any decision analysis.

We have also left aside the morbidity of surgery, which may be considerable when ureteric dissection and lymphadenectomy is carried out. Fistulae, for example, occur in nearly 3% of cases (Shingleton & Orr, 1983) but surgical complications temporarily reduce life quality and play a trivial part in the final equation dominated by survival and fertility. The final limitation of decision analysis involves our ability to measure the extent to which patients would sacrifice the chances of cure in order to avoid operative death (or vice versa), or to avoid infertility. When obtaining utilities for outcomes the ranking scale is conceptually easier for subjects to use (Gafni & Torrance, 1984), but this does not produce numerically accurate and definitive answers (Thornton, 1990). However our findings are confirmed by other reports (McNeil *et al.*, 1981; 1982) suggesting that most people prefer delayed death to immediate surgical death. The utility values obtained from the standard gamble method lie close to the range of informed human choice (Torrance, 1986) but there are still several reasons why findings may not accurately reflect subjects' true preferences:- (i) there is a tendency for individuals to exhibit pure risk aversion or a reluctance to gamble per se, (ii) practical behaviour limitations of utility assessment (Bombardier, 1982) and (iii) the difficulty subjects have in interpreting very small probabilities (Torrance, 1986). It is difficult to eliminate these errors. Furthermore it would be wrong to extrapolate treatment for individuals from population means as the optimum treatment of the disease for the mean population may not be the preferred treatment for that patient. In order to tailor the best treatment to suit each patient, sensitivity analysis was carried out to determine the effect of a range of possible utilities for cancer death, surgical death and infertility on choice of therapy. Table III shows a sensitivity analysis in which we ignore infertility, as one would when treating a patient who had 'completed her family'. When subjects are indifferent between cancer and surgical death, the results are the same as those where we wished simply to maximise chances of survival. However even if patients express a strong wish to avoid either a surgical or cancer death the optimum treatment is unchanged. This shows that our conclusions are very robust to changes in an individual's preference for surgical and cancer death and implies that patients preferences on their method of death are unlikely to change the decision.

The situation is, however, quite different when a range of utility values for infertility are added to the analysis. If we assume utilities for peri-operative and cancer death of 0.16 and 0.106 respectively (the mean values given by our subjects) and break down the analysis to include different risks of cancer spread these results are very sensitive to changes in the utility of infertility. Thus, when the utility of infertility is 95%, conservative therapy remains the preferred option for all microinvasive lesions even if the chance of spread has increased to 5% (Table IV). A utility of 0.95 implies that the subject was prepared to lower her chances of survival by 5% to preserve her reproductive function. When this view is felt less strongly but nevertheless keenly, and a patient regards life as 50 times more valuable than fertility, then a utility of 0.98 may be inferred and conservative therapy would then be appropriate for lesions less than 3 mm, but with vascular channel involvement and for lesions penetrating a depth of 3–5 mm with no vascular involvement. In other words the physician must place significant weight on the patient's desire for fertility because it has a major bearing on the choice of treatment.

Although decision analysis has limitations conventional intuitive decisions are also limited by a paucity of hard data. The advantage of decision analysis is that intuitive bias is minimised by breaking down the problem into separate components. Despite the limitations of decision analysis and the difficulty in measuring utility this is the best way to analyse the question 'how should we treat women with early microinvasive cancer of the cervix?'

### References

AVERETTE, H.E., NELSON, J.H. NG, A.B.P., HOSKINS, W.J., BOYCE, J.G. & FORD, J.H. (1976). Diagnosis and management of microinvasive (stage 1A) carcinoma of the uterine cervix. *Cancer*, (suppl. 1), **38**, 414–425.

AVERETTE, H.E. & SEVIN, B.U. (1979). Microinvasive (Stage 1A) carcinoma of the cervix. *Obstet. Gynecol. Surv.*, **34**, 833–834.

BOCCI, A., SISMONDI, P., SINISTRERO, G. & others (1981). Radio-surgical treatment of uterine cervix carcinoma at the first clinic of obstetrics and gynaecology of the University of Turin (1972–1978). *Eur. J. Gynaecol. Oncol.*, **2**, 9–16.

BOHN, J.W., KRUPP, P.J., LEE, F.Y.L. & BATSON, H.W.K. (1976). Lymph node metastasis in microinvasive epidermoid cancer of the cervix. *Obstet. Gynecol.*, **48**, 65–67.

BOMBARDIER, C., WOLFSON, A.D., SINCLAIR, A.J. & MCGEER, A. (1982). *Comparison of Three Preference Measurement Methodologies in the Evaluation of the Functional Status Index. Choices in Health Care*. Deber, R.B. & Thompson, G., (eds), University of Toronto, 145–160.

BOUTSELIS, J.G., ULLERY, J.C. & CHARME, L. (1971). Diagnosis and management of Stage 1A (microinvasive) carcinoma of the cervix. *Am. J. Obstet. Gynecol.*, **110**, 984–989.

CHITALE, A.R., BHUVANESHWARI, A.P., KILNANI, P. & PURANDARE, V.N. (1977). Pathology of microinvasive (Stage 1A) carcinoma of uterine cervix. *Indian J. Cancer*, **14**, 189–194.

CHRISTOPHERSON, W.M., GRAY, L.A. & PARKER, J.E. (1976). Microinvasive carcinoma of the uterine cervix. A long-term follow-up study of eighty cases. *Cancer*, **38**, 629–632.

CREASMAN, W.T., FETTER, B.F., CLARKE-PEARSON, D.L., KAUFMANN, L. & PARKER, R.T. (1985). Management of Stage 1A carcinoma of the cervix. *Am. J. Obstet. Gynecol.*, **153**, 164–172.

CREASMAN, W.T. & WEED, J.C. (1979). Microinvasive cancer versus occult cancer. *Radiation Oncol. Biol. Phys.*, **5**, 1871–1872.

FOUSHEE, J.H.S., GREISS, F.C. & LOCK, F.R. (1969). Stage 1A squamous cell carcinoma of the uterine cervix. *Am. J. Obstet. Gynecol.*, **105**, 46–58.

GAFNI, A. & TORRANCE, G.W. (1984). Risk attitude and time preference in health. *Management Sci.*, **30**, 440–451.

HAMBERGER, A.D., FLETCHER, G.H. & WHARTON, J.T. (1978). Results of treatment of early Stage 1 carcinoma of the uterine cervix with intracavity radium alone. *Cancer*, **4**, 980–985.

HERSHEY, J.C. & SCHOEMAKER, P.J.H. (1985). Probability versus certainty equivalence methods in utility measurement: are they equivalent? *Management Sci.*, **31**, 1213–1231.

HASUMI, K., SAKAMOTO, A. & SUGANO, H. (1980). Microinvasive carcinoma of the uterine cervix. *Cancer*, **45**, 928–931.

HSU, C.T., CHANG, Y.S. & SU, S.E. (1972). Prognosis of uterine cervical cancer with extensive lymph node metastasis. *Am. J. Obstet. Gynecol.*, **114**, 954–962.

IVERSEN, T., ABELER, V. & KNORSTAD, K.E. (1979). Factors influencing the treatment of patients with Stage 1A carcinoma of the cervix. *Br. J. Obstet. Gynecol.*, **86**, 593–597.

KRISHNA, U.R., CHITALE, A.R., AYER, B.S. & PURANDARE, V.N. (1979). Management of microinvasive carcinoma of cervix. *Indian J. Cancer*, **16**, 33–36.

LA VECCHIA, C., FRANCESCHI, S., DECARI, A., GALLUS, G., PARAZZINI, F. & MERLO, E. (1984). Invasive cervical cancer in young women. *Br. J. Obstet. Gynaecol.*, **91**, 1149–1115.

LEMAN, M.H., BESON, W.L., KURMAN, R.J. & PARK, R.C. (1976). Microinvasive carcinoma of the cervix. *Obstet. Gynecol.*, **48**, 571–578.

LOHE, K.J. (1978). Early squamous cell carcinoma of the uterine cervix. *Gynecol. Oncol.*, **6**, 10–30.

MARGULIS, R.R., ELY, C.W. & LADD, J.E. (1966). Diagnosis and management of Stage 1A (Microinvasive) Carcinoma of the cervix. *Obstet. Gynecol.*, **29**, 529–538.

MCNEIL, B.J., WELCHSELBAUM, R. & PAUKER, S.G. (1981). Speech and survival. Tradeoffs between quality and quantity of life in laryngeal cancer. *N. Engl. J. Med.*, **305**, 982–987.

MCNEIL, B.J., PAUKER, S.G., SOX, H. & TVERSKY, A. (1982). On the elicitation of preferences for alternative therapies. *N. Engl. J. Med.*, **306**, 1259–1262.

MUSSEY, E., SOULE, E.H. & WELCH, J.S. (1969). Microinvasive carcinoma of the cervix – late results of operative treatment in 91 cases. *Am. J. Obstet. Gynecol.*, **104**, 738–742.

NELSON, J.H., AVERETTE, H.E. & RICHART, R.M. (1975). *Dysplasia and early cervical cancer*. New York: Professional Education Publication, American Cancer Society.

NG, A.B.P. & REAGAN, J.W. (1969). Microinvasive carcinoma of the uterine cervix. *Am. J. Clin. Pathol.*, **52**, 511–529.

PEEL, K.R., KHOURY, G.G., JOSLIN, C.A.F., O'DONOVAN, P., MGAYA, H., KEATES, G., HEAD, C. & THOROGOOD, D.J. Cancer of the cervix in women under 40 years old: A regional survey 1975–1984. *Br. J. Obstet. Gynaecol.*, (in press).

POPKIN, D.R., PILORGE, R. & LATOUR, J.P.A. (1979). The treatment of microinvasive squamous cell carcinoma of the uterine cervix. *Gynecol. Oncol.*, **8**, 84–86.

PRZYBORA, L.A. (1965). Incipient invasion of cervix cancer; morphological aspects of carcinogenisis in 74 cases. *Gynaecologia*, **160**, 160–169.

RE, F.DI & LUCIANI, L. (1977). Clinical management of cervical carcinoma *in situ* and microinvasive carcinoma. *Arch. Geschwulstforsch.*, **47**, S346–S354.

ROCHE, W.D. & NORRIS, H.J. (1975). Microinvasive cancer of the cervix – the significance of lymphatic invasion and confluent patterns of stromal growth. *Cancer*, **36**, 180–186.

RUBIO, C.A., SODERBERG, G. & EINHORN, N. (1974). Histological and follow-up studies in cases of microinvasive carcinoma of the uterine cervix. *Acta Path. Microbiol. Scand [A]*, **82**, 397–410.

RUCH, R.M., PITCOCK, J.A. & RUCH, W.A. (1976). Microinvasive carcinoma of the cervix. *Am. J. Obstet. Gynecol.*, **125**, 87–92.

SEDLIS, A., SALL, S. & TSUKADA, Y. Microinvasive carcinoma of the uterine cervix: a clinical-pathologic study. *Am. J. Obstet. Gynecol.*, **133**, 64–74.

SESKI, J.C., ABELL, M.R. & MORLEY, G.W. (1977). Microinvasive squamous carcinoma of the cervix: Definition, histologic analysis, late results of treatment. *Obstet. Gynecol.*, **50**, 410–414.

SHINGLETON, H.M. & ORR, J.W. (1983). Cancer of the cervix. *Current review in Obstet. Gynecol.*, H.S. Churchill Livingstone: Edinburgh, London 77–84.

SIMON, N.L., GORE, H., SHINGLETON, H.M., SOONG, S.J., ORR, J. & HATCH, K.D. (1986). Study of superficially invasive carcinoma of the cervix. *Obstet. Gynecol.*, **68**, 19–24.

TAKI, I., SUGINORI, H., MATSUYAMA, T., KASHIMURA, Y. & YOSHINO, T. (1979). Treatment of microinvasive carcinoma. *Obstet. Gynecol. Surv.*, **34**, 839–840.

TARKINGTON, C.N., TWEEDDALE, D.N. & RODDICK, J.W. (1969). Microinvasive carcinoma of the cervix. *Southern Med. J.*, **62**, 1000–1002.

THOMPSON, W. (1968). Microinvasive carcinoma of the uterine cervix. *J. Arkansas Med. Soc.*, **65**, 139–143.

THORNTON, J.G. (1989). Measuring patients values in reproductive medicine. *Contemporary Rev. Obstet. & Gynaecol.*, **1**, 5–12.

TORRANCE, G.W. (1986). Measurement of health status utilities for economic appraisal: a review. *J. Health Econ.*, **5**, 1–30.

ULLERY, J.C., BOUTSELIS, J.G. & DOTSCHNER, A.C. (1965). Microinvasive carcinoma of the cervix. *Obstet. Gynecol.*, **26**, 866–875.

VAN NAGELL, J.R., GREENWELL, N., POWELL, D.F., DONALDSON, E.S., HANSON, M.B. & GAY, E.C. (1983). Microinvasive carcinoma of the cervix. *Am. J. Obstet. Gynecol.*, **145**, 981–991.

VON NEUMANN, J. & MORGENSTERN, O. (1947). *Theory of Games and Economic Behaviour*. Princetown University Press, 2nd Ed., 1953.

WEINSTEIN, M.C. (1986). Risky Choices in Medical Decision Making: A Survey. *The Green Papers on Risk on Insurance*, **11**, 197–216.

WEINSTEIN, M.C. & FINEBERG, H.V. (1980). *Clinical Decision Analysis*. Philadelphia USA: W.B. Saunders, 1980.

WILKINSON, E.J. & KOMOROWSKI, R.A. (1978). Borderline microinvasive carcinoma of the cervix. *Obstet. Gynecol.*, **51**, 472–476.

YAJIMA, A. & NODA, K. (1979). The results of treatment of microinvasive carcinoma (Stage 1A) of the uterine cervix by means of simple and extended hysterectomy. *Am. J. Obstet. Gynecol.*, **135**, 685–688.